# 

**Published:** 1874-01

**Authors:** 


Vol. IV. JANUARY, 1874. No. 1.

THE SOUTHERN

### MEDICAL RECORD:

A MONTHLY JOURNAL OF

### PRACTICAL MEDICINE.

SURGEON GENERAL'S OFFICE. LIBRARY.

EDITORS:

T. S. POWELL, M.D., - - - - - ATLANTA, GEORGIA.
W. T. GOLDSMITH, M.D., - - - - ATLANTA, GEORGIA.
T. CURTIS SMITH, M.D., - - - - MIDDLEPORT, OHIO.
SWAN M. BURNETT, M.D., - - - KNOXVILLE, TENNESSEE.
ALBAN S. PAYNE, M.D., - - - - MARKHAM'S, VIRGINIA.
A. R. KILPATRICK, M.D., - - - NAVASOTA, TEXAS.
R. C. WORD, M.D., - - - - - DECATUR, GEORGIA.

*"QUICQUID PRÆCIPIES ESTO BREVIS."*

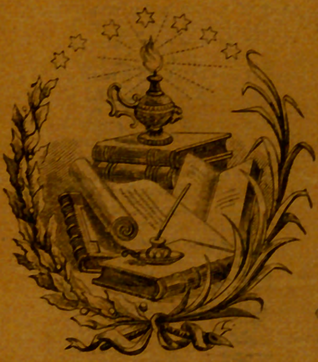

S.G.O. 60535

ATLANTA, GEORGIA:
SOUTHERN PUBLISHING COMPANY, PRINTERS.
1874.

TERMS, $2 50 PER ANNUM, IN ADVANCE. SINGLE NUMBER, 25 CENTS.

